# SARS-CoV-2 Viral Shedding and Rapid Antigen Test Performance — Respiratory Virus Transmission Network, November 2022–May 2023

**DOI:** 10.15585/mmwr.mm7316a2

**Published:** 2024-04-25

**Authors:** Sarah E. Smith-Jeffcoat, Alexandra M. Mellis, Carlos G. Grijalva, H. Keipp Talbot, Jonathan Schmitz, Karen Lutrick, Katherine D. Ellingson, Melissa S. Stockwell, Son H. McLaren, Huong Q. Nguyen, Suchitra Rao, Edwin J. Asturias, Meredith E. Davis-Gardner, Mehul S. Suthar, Hannah L. Kirking, RVTN-Sentinel Study Group, RVTN-Sentinel Study Group, Melissa A. Rolfes, Jessica E. Biddle, Yuwei Zhu, Karla Ledezma, Kathleen Pryor, Ellen Sano, Joshua G. Petrie

**Affiliations:** ^1^Coronavirus and Other Respiratory Viruses Division, National Center for Immunization and Respiratory Diseases, CDC; ^2^Influenza Division, National Center for Immunization and Respiratory Diseases, CDC; ^3^Vanderbilt University Medical Center, Nashville, Tennessee; ^4^University of Arizona Colleges of Medicine and Public Health, Tucson, Arizona; ^5^Division of Child and Adolescent Health, Department of Pediatrics, Vagelos College of Physicians and Surgeons, Columbia University, New York, New York; ^6^Department of Population and Family Health, Mailman School of Public Health, New York, New York; ^7^New York-Presbyterian Hospital, New York, New York; ^8^Department of Emergency Medicine, Vagelos College of Physicians and Surgeons, Columbia University, New York, New York; ^9^Marshfield Clinic Research Institute, Marshfield, Wisconsin; ^10^Children’s Hospital Colorado, Aurora, Colorado; ^11^Department of Pediatrics-Infectious Diseases, Emory Vaccine Center, Emory Primate Research Center, Emory University School of Medicine, Atlanta, Georgia.; National Center for Immunization and Respiratory Diseases, CDC; National Center for Immunization and Respiratory Diseases, CDC; Vanderbilt University Medical Center, Nashville, Tennessee; University of Arizona, Tucson, Arizona; University of Arizona, Tucson, Arizona; Columbia University Irvin Medical Center, New York, New York; Marshfield Clinic Research Institute, Marshfield, Wisconsin

**Summary**

**What is already known about this topic?**

During the COVID-19 pandemic, rapid antigen tests were found to detect potentially transmissible SARS-CoV-2 infection, but antigen tests were less sensitive than reverse transcription–polymerase chain reaction (RT-PCR) testing.

**What is added by this report?**

During November 2022–May 2023, among persons infected with SARS-CoV-2, sensitivity of rapid antigen tests was 47% compared with RT-PCR and 80% compared with viral culture. Antigen tests continue to detect potentially transmissible infection but miss many infections identified by positive RT-PCR test results.

**What are the implications for public health practice?**

Rapid antigen tests can aid in identifying infectiousness of persons infected with SARS-CoV-2 and providing access to diagnostic testing for persons with COVID-19 symptoms. Persons in the community eligible for antiviral treatment should seek more sensitive diagnostic tests from a health care provider. Clinicians should consider RT-PCR testing for persons for whom antiviral treatment is recommended.

## Abstract

As population immunity to SARS-CoV-2 evolves and new variants emerge, the role and accuracy of antigen tests remain active questions. To describe recent test performance, the detection of SARS-CoV-2 by antigen testing was compared with that by reverse transcription–polymerase chain reaction (RT-PCR) and viral culture testing during November 2022–May 2023. Participants who were enrolled in a household transmission study completed daily symptom diaries and collected two nasal swabs (tested for SARS-CoV-2 via RT-PCR, culture, and antigen tests) each day for 10 days after enrollment. Among participants with SARS-CoV-2 infection, the percentages of positive antigen, RT-PCR, and culture results were calculated each day from the onset of symptoms or, in asymptomatic persons, from the date of the first positive test result. Antigen test sensitivity was calculated using RT-PCR and viral culture as references. The peak percentage of positive antigen (59.0%) and RT-PCR (83.0%) results occurred 3 days after onset, and the peak percentage of positive culture results (52%) occurred 2 days after onset. The sensitivity of antigen tests was 47% (95% CI = 44%–50%) and 80% (95% CI = 76%–85%) using RT-PCR and culture, respectively, as references. Clinicians should be aware of the lower sensitivity of antigen testing compared with RT-PCR, which might lead to false-negative results. This finding has implications for timely initiation of SARS-CoV-2 antiviral treatment, when early diagnosis is essential; clinicians should consider RT-PCR for persons for whom antiviral treatment is recommended. Persons in the community who are at high risk for severe COVID-19 illness and eligible for antiviral treatment should seek testing from health care providers with the goal of obtaining a more sensitive diagnostic test than antigen tests (i.e., an RT-PCR test).

## Introduction

SARS-CoV-2 rapid antigen tests were developed and received Food and Drug Administration Emergency Use Authorization early during the COVID-19 pandemic.* These tests were initially rolled out broadly in the United States to diagnose cases and isolate persons who received positive test results to aid in preventing onward spread at a time when population SARS-CoV-2 immunity was low, and rates of severe COVID-19–associated outcomes were high. In addition, demands for testing exceeded supply, and long turnaround times for reverse transcription–polymerase chain reaction (RT-PCR) test results contributed to ongoing transmission. Wide access to antigen tests was made possible through U.S. government initiatives implemented to prevent transmission.^†^^,^^§^ After the emergence of the Omicron variant in late 2021, at-home antigen test use began to increase sharply (*1*,*2*).

Studies conducted during circulation of SARS-CoV-2 pre-Delta and Delta variants illustrated that antigen tests have high specificity, but lower sensitivity when compared with RT-PCR tests, thereby missing a substantial number of infections but correlating more closely with viral culture results (*3*–*6*). Viral culture, although not frequently used for routine patient care, is able to detect actively replicating virus (thus identifying when a person is likely to be infectious), whereas RT-PCR cannot distinguish between replicating virus and viral fragments. Most of these studies included few participants with vaccine- or infection-induced immunity. SARS-CoV-2 variants and population immunity have evolved since many of the studies assessing antigen tests were performed; thus, the role that antigen tests should play in diagnosing SARS-CoV-2 infection remains an active question. The objective of this investigation was to reevaluate the performance characteristics of SARS-CoV-2 antigen tests with those of RT-PCR and viral culture tests during a period with greater population immunity and more recently circulating SARS-CoV-2 Omicron variants.

## Methods

This evaluation included participants enrolled in an antigen test substudy within a case-ascertained household transmission study during November 2022–May 2023^¶^ (*7*). Index patients with confirmed SARS-CoV-2 infection and their household contacts were enrolled within 7 days of illness onset in the index patient. Participants completed baseline surveys including demographic characteristics, COVID-19 signs or symptoms (symptoms),** vaccination,^††^ and self-reported previous infection. Participants (index patients and contacts) also provided a blood specimen for SARS-CoV-2 anti-N antibody detection^§§^ (*8*,*9*). For 10 days after enrollment, all participants completed daily COVID-19 symptom diaries and collected two nasal swabs each day. One swab was self-collected in viral transport media, stored in refrigerator for up to 72 hours, then collected by a study team member and stored at −12°F (−80°C) until aliquoted for automated RT-PCR (Hologic Panther Fusion)^¶¶^ and viral culture,*** and the other swab was used for at-home antigen testing.^†††^ Participants interpreted and reported their antigen test results in their daily symptom diary. For this analysis, SARS-CoV-2 infection was defined as at least one positive RT-PCR test result during the study period; onset was defined as the first day of symptoms or, if the participant remained asymptomatic, day of first positive test result.

Among participants who ever received a positive RT-PCR test result and had one or more paired RT-PCR and antigen results reported, the percentage of positive antigen, RT-PCR, and viral culture results was calculated for each day relative to onset. The percentage of positive antigen test results was stratified by symptom and fever status. Sensitivity of antigen testing among paired samples collected from 2 days before until 10 days after onset was computed using two references: 1) same-day positive RT-PCR result and 2) same-day positive culture result, stratified by overall symptom status and presence of fever alone or fever or cough. Wilson score intervals were used for calculating 95% CIs around percentage of positive test results. Cluster-robust bootstrapping was used to calculate 95% CIs around sensitivity to account for within-participant correlation. All analyses were performed in RStudio (version 4.2.3; RStudio). This study was reviewed and approved by the Vanderbilt University Institutional Review Board.^§§§^

## Results

### Characteristics of Study Participants

Among 354 participants in 129 households, 236 (67%) received a positive SARS-CoV-2 RT-PCR test result and were included in this investigation (Table). Participants ranged in age from 2 months to 83 years (median = 36 years; IQR = 17–50 years), 133 (56%) were non-Hispanic White persons, and 140 (59%) were female. Ninety-two (40%) participants reported receipt of a COVID-19 vaccine ≤12 months before enrollment; 82 (35%) had received ≥2 doses, but the most recent dose was >12 months before enrollment; 57 (24%) were unvaccinated (including those who had only ever received 1 dose); and vaccination status was unknown for five participants. A total of 102 (43%) participants had self-reported or serologic evidence of previous SARS-CoV-2 infection. At least one COVID-19 symptom was reported by 219 (93%) participants, including 182 (77%) who reported cough and 156 (66%) who reported fever.

**TABLE:** Characteristics of participants infected with SARS-CoV-2^*^ (N = 236) — Respiratory Virus Transmission Network, November 2022–May 2023

Characteristic	No. (%)
**Age at enrollment, yrs, median (IQR)**	36 (17–50)
**Age group, yrs**	
0–4	22 (9)
5–11	15 (6)
12–17	23 (10)
18–49	114 (48)
50–64	44 (20)
≥65	18 (7)
**Gender**	
Female	140 (59)
Male	95 (40)
Nonbinary/Transgender	1 (<1)
**Race and ethnicity^†^**	
Black or African American	17 (7)
White	133 (57)
Hispanic or Latino	69 (29)
Other	14 (6)
Unknown/Refused	3 (1)
**SVI, median (IQR)^§^**	0.43 (0.19–0.80)
**Any chronic medical condition**	110 (47)
**Vaccination status** ^¶^	
Unvaccinated	57 (24)
Vaccinated >12 mos before enrollment	82 (35)
Vaccinated ≤12 mos before enrollment	92 (40)
Unknown	5 (<1)
Any previous SARS-CoV-2 infection**	102 (43)
**Any COVID-19 symptoms** ^††^	219 (93)
Any cough	182 (77)
Any fever	156 (66)
One or more positive viral cultures	143 (61)
One or more positive antigen tests	164 (69)

**Abbreviations:** RT-PCR = reverse transcription–polymerase chain reaction; SVI = Social Vulnerability Index.

* SARS-CoV-2 infection defined as having received at least one positive RT-PCR result during study testing.

^†^ Persons of Hispanic or Latino (Hispanic) origin might be of any race but are categorized as Hispanic; all racial groups are non-Hispanic.

^§^ SVI was determined using the 2020 U.S. Census Bureau decennial tract location of the home. SVI uses 16 census variables to indicate the relative vulnerability of every census tract to a hazardous event with values closer to 1 representing highly vulnerable areas and values closer to 0 representing least vulnerable areas.

^¶^ Vaccination history was self-reported and then verified by study team. Participants were considered vaccinated within 12 months before enrollment if they had received ≥2 doses and the most recent dose was received between 14 days and 12 months before enrollment; vaccinated >12 months before enrollment if they had received ≥2 doses and the most recent dose was received >12 months before enrollment; and unvaccinated if they received <2 doses before enrollment.

** By self-report or serologic evidence. Previous SARS-CoV-2 infection was defined as self-report of a previous infection ≥1 month before enrollment or by detection of antinucleocapsid antibodies from a dried blood spot collected at baseline.

^††^ Elicited COVID-19 signs and symptoms included fever (including feeling feverish or chills), cough, sore throat, runny nose, nasal congestion, fatigue (including feeling run-down), wheezing, trouble breathing (including shortness of breath), chest tightness (including chest pain), loss of smell or loss of taste, headache, abdominal pain, diarrhea, vomiting, and muscle or body aches.

### SARS-CoV-2 Test Results

Among the 236 SARS-CoV-2–infected participants (i.e., those who received a positive RT-PCR test result), 2,244 antigen results were reported and included in analyses. Overall, 143 (61%) participants received one or more positive culture result, and 164 (69%) received one or more positive antigen test result.

**FIGURE 1:**
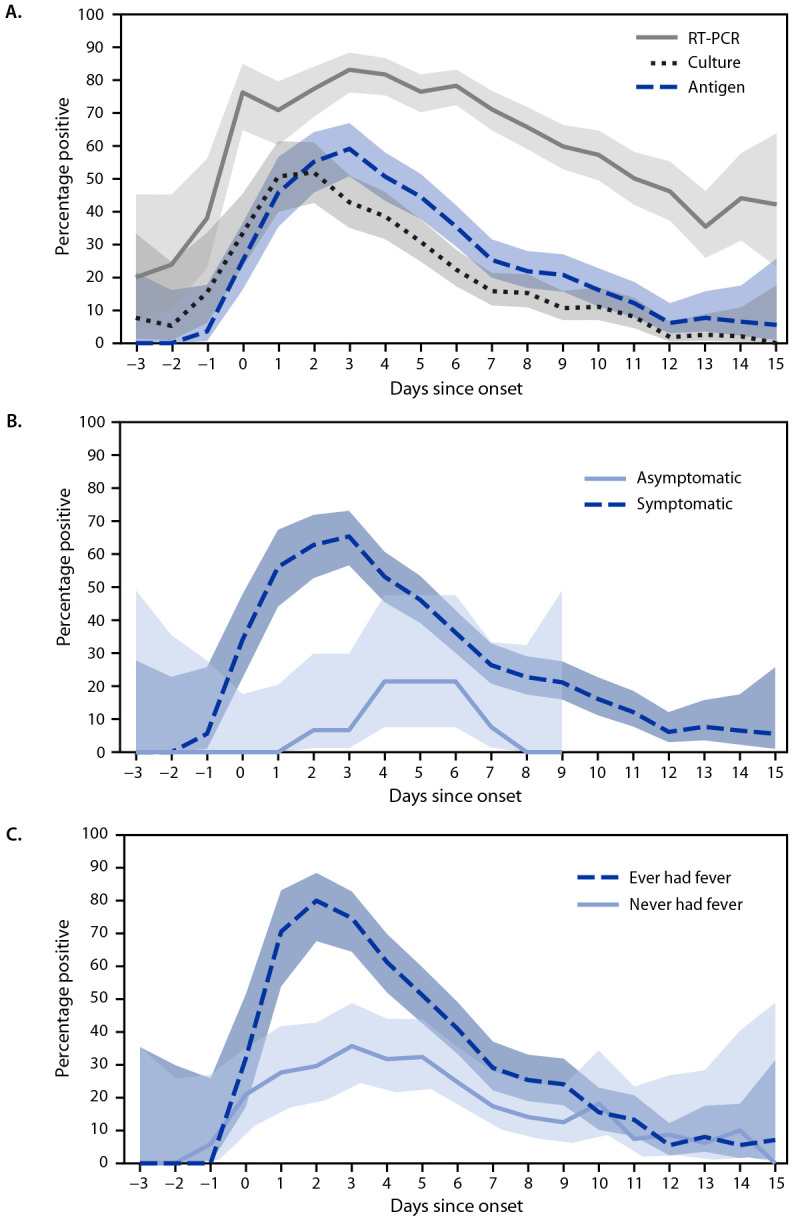
Percentage* of rapid antigen, reverse transcription–polymerase chain reaction, and viral culture test results that were positive for SARS-CoV-2 (A) and percentage of antigen test results that were positive, by symptom status† (B) and presence of fever (C) each day since onset^§^ among participants infected with SARS-CoV-2^¶^ — Respiratory Virus Transmission Network, November 2022–May 2023 **Abbreviation:** RT-PCR = reverse transcription–polymerase chain reaction. * With 95% CIs indicated by shaded areas. ^†^ Elicited COVID-19 signs and symptoms included fever (including feeling feverish or chills), cough, sore throat, runny nose, nasal congestion, fatigue (including feeling run-down), wheezing, trouble breathing (including shortness of breath), chest tightness (including chest pain), loss of smell or loss of taste, headache, abdominal pain, diarrhea, vomiting, and muscle or body aches. ^§^ Date of symptom onset or, for asymptomatic persons, date of first positive test result. ^¶^ SARS-CoV-2 infection defined as having received at least one positive RT-PCR test result during study testing.

The highest percentage of positive antigen (59%; 95% CI = 51%–67%) and RT-PCR (83%; 95% CI = 76%–88%) test results occurred 3 days after onset (Figure 1). The highest percentage of positive viral culture results (52%; 95% CI = 43%–61%) occurred 2 days after onset. Among the 219 symptomatic participants, the highest percentage of positive antigen test results was 65% (95% CI = 57%–73%) at 3 days after onset among those who experienced any COVID-19 symptom and 80% (95% CI = 68%–88%) at 2 days after onset among those who reported fever.

### Sensitivity of Antigen Testing

**FIGURE 2:**
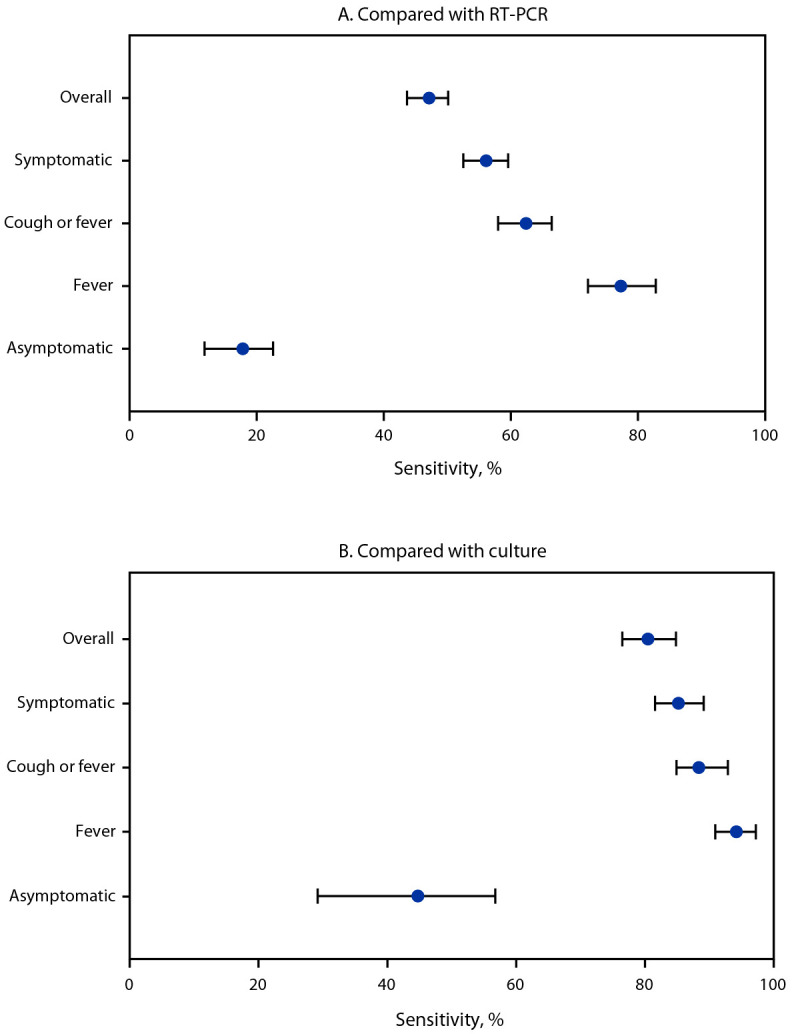
Sensitivity* of rapid antigen tests results for diagnosing SARS-CoV-2 infection compared with reverse transcription–polymerase chain reaction (A) and viral culture (B), overall and by presence of symptoms^†^ — Respiratory Virus Transmission Network, November 2022–May 2023 **Abbreviation:** RT-PCR = reverse transcription–polymerase chain reaction. * With 95% CIs indicated by error bars. ^†^ Elicited COVID-19 signs and symptoms included fever (including feeling feverish or chills), cough, sore throat, runny nose, nasal congestion, fatigue (including feeling run-down), wheezing, trouble breathing (including shortness of breath), chest tightness (including chest pain), loss of smell or loss of taste, headache, abdominal pain, diarrhea, vomiting, and muscle or body aches.

Compared with same-day collected RT-PCR and culture results, the overall sensitivities of daily antigen test results were 47% (95% CI = 44%–50%) and 80% (95% CI = 76%–85%), respectively (Figure 2) (Supplementary Table, https://stacks.cdc.gov/view/cdc/153544). When stratified by symptoms experienced on the day of specimen collection, antigen test sensitivity increased with occurrence of any COVID-19 symptoms (56% and 85% compared with RT-PCR and culture, respectively) and peaked on days that fever was reported (77% and 94% compared with RT-PCR and culture, respectively). Compared with RT-PCR and culture results, sensitivity of antigen testing was low on days when no symptoms were reported (18% and 45%, respectively).

## Discussion

Among participants enrolled in a household transmission study during a period of increased disease- and vaccine-induced immunity, and when circulating viruses differed antigenically from the ancestral SARS-CoV-2 strain, antigen and culture tests detected a similar proportion of SARS-CoV-2 infections, but detection by RT-PCR was higher than that by either antigen or culture. Similarly, paired antigen test sensitivity was low compared with RT-PCR (47%), but relatively high compared with culture (80%). The sensitivity of antigen testing was higher when symptoms were present on the test day and peaked on days when participants reported fever. Although viral culture is not an absolute marker of transmissibility, this pattern suggests that positive antigen test results could indicate transmissible virus; thus, antigen tests might aid persons with COVID-19 in determining when they are no longer infectious once symptoms begin to resolve.

The findings from this investigation remain similar to those reported in other studies throughout the COVID-19 pandemic (*3*–*6*). For example, considering the current study’s sensitivity results, an early 2021 study comparing antigen testing with RT-PCR and culture found similar antigen test sensitivity compared with culture (84%), but slightly higher sensitivity compared with RT-PCR (64%) (*3*). The sensitivity difference between these two studies could be attributed to many factors, including differences in participant immunity, infecting variants, the limit of detection of the reference RT-PCR, or sampling methods.

Minimizing false negative test results is important because additional modalities, including antiviral medications, are available to prevent severe outcomes. Antiviral treatments for SARS-CoV-2 infection should be started as soon as possible, and within 5–7 days of symptom onset.^¶¶¶^ Therefore, persons who are at higher risk for severe illness and eligible for antiviral treatment would benefit from a more accurate diagnostic test. In most clinical scenarios in the United States, this approach means a SARS-CoV-2 RT-PCR test would be a better diagnostic test to minimize the risk for a false-negative result. Alternatively, if RT-PCR tests are not available or accessible, clinicians and patients should follow FDA’s serial antigen testing recommendations to help optimize diagnostic test performance.****

### Limitations

The findings in this report are subject to at least three limitations. First, participants included in this analysis might not represent all U.S. persons infected with SARS-CoV-2 and represent those with mild to moderate illness. These findings might not apply to persons with more severe COVID-19 illness. Second, one commercially available antigen test was used in this study; results might not apply to all available antigen tests. Finally, because of the parent study design, onset for asymptomatic participants (i.e., the day of the first positive test result), could be biased if household members were not enrolled early enough to record the earliest positive test result.

### Implications for Public Health Practice

As COVID-19 becomes endemic and public focus shifts from stopping transmission to preventing severe illness,^††††^ diagnostic testing should emphasize use of the best tests to identify infection in persons who would benefit from treatment. The low sensitivity of antigen testing among persons with asymptomatic infections illustrates that these tests should only be used once symptoms are present. Conversely, the higher sensitivity when symptoms are present (especially cough or fever) supports the need to stay at home when symptomatic, irrespective of test result.^§§§§^ The low sensitivity of antigen tests compared with RT-PCR tests has implications for timely initiation of anti–SARS-CoV-2 treatment when early and accurate diagnosis is important. With several treatment options available, clinicians should consider more sensitive RT-PCR tests for accurate diagnosis in persons at higher risk for severe illness to minimize delays in treatment initiation. Persons in the community who are at high risk for severe COVID-19 illness and eligible for antiviral treatment should seek testing from health care providers with the goal of obtaining a more sensitive diagnostic test than antigen tests (i.e., an RT-PCR test).

## Footnotes

*
https://www.fda.gov/news-events/press-announcements/coronavirus-covid-19-update-fda-authorizes-first-antigen-test-help-rapid-detection-virus-causes

†
https://www.covid.gov/tools-and-resources/resources/tests

§
https://www.whitehouse.gov/wp-content/uploads/2021/01/National-Strategy-for-the-COVID-19-Response-and-Pandemic-Preparedness.pdf

¶ The Respiratory Virus Transmission Network sites that participated in the antigen substudy were located in Arizona, Colorado, New York, Tennessee, and Wisconsin. Persons who received test results positive for SARS-CoV-2 were recruited from participating medical centers, community testing sites, actively surveilled cohorts, and public health registries at five sites.

** Elicited COVID-19 symptoms included fever (including feeling feverish and chills), cough, sore throat, runny nose, nasal congestion, fatigue (including feeling run-down), wheezing, trouble breathing (including shortness of breath), chest tightness (including chest pain), loss of smell or loss of taste, headache, abdominal pain, diarrhea, vomiting, and muscle or body aches.

†† Vaccination history was self-reported and then verified by study team using state vaccination registries, electronic medical records, and pharmacy records.

§§ Detection of antinucleocapsid antibodies from a dried blood spot collected at baseline was considered serological evidence of previous SARS-CoV-2 infection. Simultaneous detection and differentiation of total binding antibody (immunoglobulin [Ig]M, IgG, and IgA) to SARS-CoV-2 2019-nCoV WHU02 strain nucleocapsid protein, Wuhan-Hu-1 strain spike protein receptor binding domain, and Wuhan-Hu-1 strain spike protein trimer in capillary (finger stick) dried blood was performed using the ProcartaPlex Immunoassay multiplex custom panel (Invitrogen) deployed on the MAGPIX System (Luminex).

¶¶ RT-PCR results were interpreted as categorically positive or negative according to the FDA-authorized parameters of the Hologic Panther Fusion SARS-CoV-2 assay, as utilized for in vitro diagnostic purposes. https://www.fda.gov/media/136156/download?attachment

*** Viral culture was performed on Vero E6 cells expressing both ACE2 and TMPRSS2. Cells were infected with serial dilutions of virus in Dulbecco’s Modified Eagle Medium (DMEM) containing ciprofloxacin, and cytopathic effect (CPE) was visually observed during a period of 5 days. Observation of CPE was considered positive for viral culture.

††† Quidel QuickVue At-Home COVID-19 Test (available as over-the-counter). https://www.fda.gov/media/146312/download

§§§ 45 C.F.R. part 46.114; 21 C.F.R. part 56.114.

¶¶¶
https://www.cdc.gov/coronavirus/2019-ncov/your-health/treatments-for-severe-illness.html

****
https://www.fda.gov/medical-devices/safety-communications/home-covid-19-antigen-tests-take-steps-reduce-your-risk-false-negative-results-fda-safety

††††
https://www.cdc.gov/respiratory-viruses/whats-new/changing-threat-covid-19.html

§§§§
https://www.cdc.gov/respiratory-viruses/prevention/precautions-when-sick.html
